# Hierarchical Equations-of-Motion Method for Momentum System–Bath Coupling

**DOI:** 10.1021/acs.jpcb.1c02431

**Published:** 2021-04-30

**Authors:** Maxim F. Gelin, Raffaele Borrelli, Lipeng Chen

**Affiliations:** † School of Sciences, Hangzhou Dianzi University, Hangzhou 310018, China; ‡ DISAFA, University of Torino, Grugliasco I-10095, Italy; ¶ Max Planck Institute for the Physics of Complex Systems, D-01187 Dresden, Germany

## Abstract

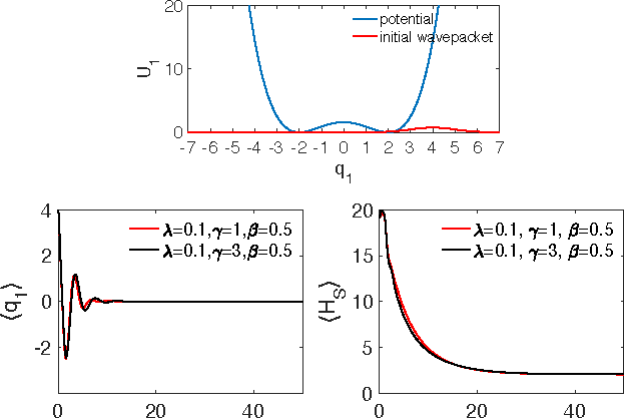

For a broad class of quantum models of practical interest, we demonstrate that the Hamiltonian of the system nonlinearly coupled to a harmonic bath through the system and bath coordinates can be equivalently mapped into the Hamiltonian of the system bilinearly coupled to the bath through the system and bath momenta. We show that the Hamiltonian with bilinear system–bath momentum coupling can be treated by the hierarchical equations-of-motion (HEOM) method and present the corresponding proof-of-principle simulations. The developed methodology creates the opportunity to scrutinize a new family of nonlinear quantum systems by the numerically accurate HEOM method.

## 1 Introduction

The hierarchical equations-of-motion (HEOM) method^1−10^ is nowadays the most powerful technique to study dissipative quantum dynamics within the system–bath approach (see ref (11) for a recent comprehensive review). HEOM permits the simulation of evolutions of various quantum systems numerically accurately, for the entire range of system–bath couplings and bath memories. Conceptually, HEOM can be applied to any system–bath problem if (i) the heat bath can be represented as a collection of noninteracting bosons, Fermions, or spins, (ii) the bath spectral density can be modeled by a linear combination of Drude–Lorenz spectral densities, and (iii) the system–bath coupling Hamiltonian is linear in the bath coordinate. The above three assumptions are well fulfilled for a large variety of quantum dissipative systems of broad interest. Yet, researchers nowadays are challenged to realistically simulate dynamics of systems of ever increasing complexity and with ever increasing accuracy. It is thus tempting to explore whether the HEOM machinery can be extended beyond postulates i–iii.

It may look unlikely that postulate i can be lifted within the HEOM paradigm, because a possibility to treat the bath dynamics analytically as well as integrate bath variables out is at the very core of the HEOM method. Nevertheless, Yan^12^ as well as Hsieh and Cao^13,14^ (gHEOM) developed a method which permits one to systematically take into account higher-order cumulants of the bath influence functional. Restriction ii has also been overcome recently: the so-called eHEOM methodology treats arbitrary bath spectral densities by using the Fourier, Gauss–Legendre, or Chebyshev-quadrature spectral decomposition.^15−27^ As for assumption iii, there exist stochastic^28^ and density-matrix-based^29,30^ methods allowing one to treat system–bath coupling which is quadratic in system–bath coordinates.

In the present work, we demonstrate how assumption iii can be lifted for a broad class of problems of practical interest and formulate the HEOM methodology toward system–bath couplings which are nonlinear in both system and bath coordinates. More specifically, we consider a microscopic model of energy transport in molecular chains with nearest-neighbor interactions, which is ubiquitous in the study of heat-transfer and vibrational energy redistribution. We assume that the first particle in the chain (the system) interacts with its neighbor via an arbitrary (nonlinear) pair potential, while all other particles in the chain (the bath) interact with each other via nearest-neighbor harmonic potentials. In sections 2 and 3 we show how the Hamiltonian of this chain model can be transformed into the system–bath form suitable for the application of the HEOM formalism, where the system–bath coupling Hamiltonian is linear both in the system and in the bath momentum operators. The corresponding HEOM equations are derived in section 4. In section 5, the obtained momentum HEOM scheme is applied for the simulation of two models of general interest which can help to understand the fundamental role of the momentum coupling operator in relaxation processes. The main results are briefly summarized in section 6. Technical derivations are deferred to Appendixes A and B. Possible extensions of the momentum HEOM are discussed in Appendix C.

## 2 Starting Equations

Let us consider a chain of *N* point particles of masses *m*_*k*_ with positions *X*_*k*_ and momenta *P*_*k*_ coupled by nearest-neighbor nonlinear potentials *U*_*k*_(*X*_*k*+1_ – *X*_*k*_). The chain is described by the Hamiltonian
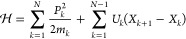
1If *U*_*k*_(*X*) are polynomials up to the forth-order in *X*, eq 1 corresponds to a quantum version of the celebrated Fermi–Pasta–Ulam model^31,32^ or Bose–Hubbard model.^33,34^ If all particles are the same and are all coupled by Hookean springs, the Hamiltonian of eq 1 yields the so-called Rubin model.^35,36^ The Hamiltonian of eq 1 is also frequently used for the description of quantum dynamics in terms of hierarchies of effective modes.^37,38^

Let us now introduce the new coordinates, the center-of-mass coordinate
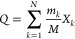
2( is the total mass) and the relative distance coordinates
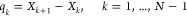
3The coordinate transformation of eqs 2 and 3 is linear, but not canonical, while the determinant of the transformation matrix equals one.^39−41^ The original momenta *P*_*k*_ are connected to the new momenta *P* ≡–*iℏd*/*dQ* and *p*_*k*_ ≡–*iℏd*/*dq*_*k*_ as follows:
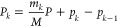
4In these new variables
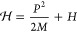
5where
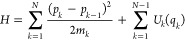
6(*p*_0_ ≡ *p*_*N*_ ≡ 0). The first term in eq 5 describes the center-of-mass motion of the chain, which is totally decoupled from the internal dynamics of the chain specified by the Hamiltonian *H*. Interestingly, there is no position couplings in *H*. Instead, the neighboring oscillators are bilinearly coupled through their momenta. Such momentum couplings are inevitable e.g. in molecular physics, when molecules are modeled as a collection of interacting atoms and the nuclear center-of-mass motion is removed.^42−44^ In addition, bilinear coordinate and momentum system–bath couplings naturally arise after the unitary transformation of the exciton-vibrational Hamiltonian with standard coordinate couplings, allowing the alternative formulation of HEOM through an effective Fokker–Planck equation.^7^ Generally speaking, momentum couplings are not limited to the chain model described above. For example, two-particle couplings ∼ *p*_*k*_*p*_*k*′_, where *k*, *k*′ label any two vibrational degrees of freedom, can be at the origin of Fermi-resonances (and of energy transfer in general) in molecular systems described in curvilinear coordinates.^45,46^

## 3 System–Bath Approach for Momentum Coupling

Let us assume that the particle 1 is the system and the remaining particles form a bath. Then we recast the Hamiltonian of eq 6 in the system–bath form
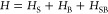
7Here
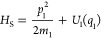
8is the system Hamiltonian
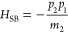
9is the system–bath coupling Hamiltonian
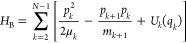
10is the bath Hamiltonian, and
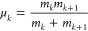
11are the reduced masses of the bath oscillators (μ_1_ = *m*_1_, μ_*N*–1_ = *m*_*N*–1_). Now assume that the bath is harmonic
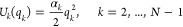
12(remind that *U*_1_(*q*_1_) can be arbitrary). The so-obtained Hamiltonian has been used for the studies of energy transfer in molecular chains by classical molecular dynamics and quantum model simulations,^47^ path-integral methods,^48^ and mixed quantum-classical simulations.^49^

It is convenient to introduce the dimensionless bath coordinates and momenta
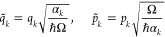
13where Ω is a certain characteristic harmonic frequency. Then
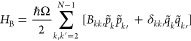
14where
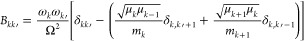
15and
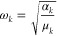
16are the harmonic frequencies of the bath oscillators. *B*_*kk*′_ is a symmetric matrix which can be diagonalized by the orthogonal transformation
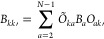
17where *B*_*a*_ are the eigenvalues of *B*_*kk*′_. Introducing the new variables
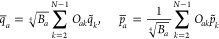
18we recast the bath Hamiltonian into a standard form describing a collection of normal modes
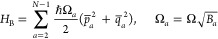
19The total Hamiltonian is thus given by eq 7, where the system Hamiltonian is determined by eq 8, the bath Hamiltonian is specified by eq 19, and the system–bath coupling is described by
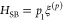
20where the collective bath momentum ξ^(*p*)^ is defined as
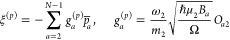
21It is appropriate to mention that system–bath Hamiltonians with momentum coupling and mixed coordinate–momentum couplings are well-known in the literature, where different (*p*_1_*p̅*_*a*_, *q*_1_*p̅*_*a*_, *p*_1_*q̅*_*a*_, *q*_1_*q̅*_*a*_) couplings and combinations thereof are considered. According to Leggett’s terminology, coordinate and momentum couplings cause normal and anomalous dissipation, correspondingly,^50^ and combined influence of normal and anomalous dissipation has been scrutinized for various harmonic systems.^50−58^ Charged oscillators in magnetic field have been considered in ref (59), dynamics of classical spins have been studied in ref (60), and Kramers’ problem in the presence of coordinate and momentum couplings has been investigated in refs (61 and 62). However, all these position–momentum couplings were introduced in an ad hoc manner, and to our knowledge, harmonic systems were considered only. In the present work, the momentum system–bath coupling arises naturally from the physically motivated Hamiltonian with (nonlinear) coordinate coupling as a result of the removal of the center-of-mass motion. The momentum coupling is thereby caused by purely geometric effects, i.e., by the transformation of variables via eqs 2 and 3.

## 4 Construction of the Momentum HEOM

Let us consider the system dynamics driven by the total Hamiltonian *H* of eq 7 defined via eqs 8, 19, and 20. The corresponding Liouville–von Neumann equation for the total density matrix ρ(*t*) reads
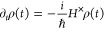
22where *A*^×^*f* = *Af* – *fA* for any operator *f*. Changing to the interaction representation with respect to *H*_B_, we obtain
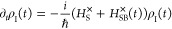
23where
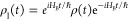
24and
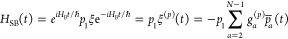
25Here the time dependence of the bath momentum coupling operator ξ^(*p*)^(*t*) and of the bath momenta *p̅*_*a*_(*t*) is generated exclusively by the bath Hamiltonian *H*_B_. Obviously, ξ^(*p*)^(*t*) is a stationary quantum Gaussian non-Markovian process whose stochastic properties are fully specified by the momentum coupling correlation function
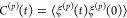
26By introducing the momentum bath spectral density
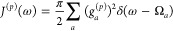
27and changing to creation and annihilation operators , , we obtain the familiar formula
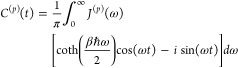
28where β = 1/*k*_B_*T* is the inverse temperature. Interestingly, *C*^(*p*)^(*t*) ≡ *C*^(*q*)^(*t*), where *C*^(*q*)^(*t*) = ⟨ξ^(*q*)^(*t*)ξ^(*q*)^(0)⟩ and ξ^(*q*)^ is the collective coordinate operator obtained by replacing *p̅*_*a*_ → *q̅*_*a*_ in eq 21.

The above considerations make it clear that all general methods of construction of HEOM for harmonic baths linearly coupled to the system through the bath coordinates can be directly applied to the case of linear coupling of the system through the bath momenta.^9^ To obtain the explicit form of the HEOM equations, we need to specify the bath spectral density (see Appendix A for the discussion of the choice of *J*^(*p*)^(ω)). In the present work, we adopt a Drude spectral density^36^
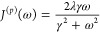
29where λ is the system–bath coupling and γ is the inverse bath correlation (memory) time. By applying the Padé spectral decomposition^63,64^ and employing the identity
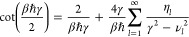
30the correlation function for the Drude spectral density can be calculated as
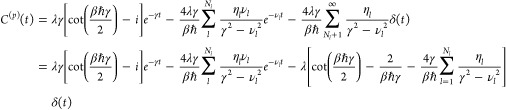
31where η_*l*_ and ν_*l*_ are the prefactor and frequency of the *l*th Padé term. Because the correlation function is a linear combination of exponentials, HEOM can be derived in the usual way. If we write the momentum coupling correlation function as
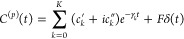
32we obtain the conventional form of HEOM for the auxiliary density operators
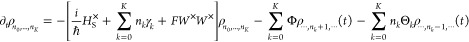
33where Φρ = *i*[*W*, ρ], , , and the auxiliary density operator with *n*_0_ = ··· = *n*_*K*_ = 0 corresponds to the actual reduced density operator, ρ_0_, ..., 0(*t*) ≡ ρ_S_(*t*). As distinct from the case of conventional case of coordinate system–bath coupling, we have
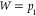
34owing to the bilinear momentum system–bath coupling.

The derivation of the momentum HEOM given above is rather formal. For better understanding of relaxation mechanisms induced by momentum coupling, it is appropriate to look at the problem from a different, more physical perspective. This is done in Appendix A, where we show that the system–bath Hamiltonian of eq 7 with bilinear momentum coupling, eqs 19 and 20, can be mapped one-to-one to the corresponding Hamiltonian with bilinear momentum-coordinate coupling, *H*_SB_ = *p*_1_ξ^(*q*)^, where ξ^(*q*)^ a linear combination of the bath coordinates. This transformation allows us to uncover physical meaning of the momentum coupling and momentum HEOM. Furthermore, the existence of this transformation proves that numerical convergence of the momentum-coupling HEOM should be similar to that of the conventional coordinate-coupling HEOM.

## 5 Illustrative Calculation

In the present section, we invoke the momentum HEOM of eq 33 to simulate the dynamics of the harmonic (section 5.1) and anharmonic double-well (section 5.2) systems. The dynamics of both models are described in dimensionless variables which are specified in sections 5.1 and 5.2. For the initial state of the momentum HEOM, we choose a Gaussian wavepacket
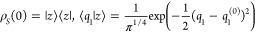
35where the initial dimensionless position is set to *q*_1_^(0)^ = 4, which corresponds to a highly nonequilibrium initial preparation of the system. The physical quantities we are interested in are the expectation values of the position operator *q*_1_ and the system Hamiltonian *H*_S_
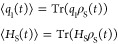
36which are evaluated for different system–bath couplings λ and inverse bath memory times γ. The counter term in the system–bath Hamiltonian is included in the simulations (see Appendix A for the details).

For the numerical simulation of eq 33, we expand the system Hamiltonian *H*_S_ and the operator *W* of eq 34 in matrix form using the harmonic oscillator basis functions. We have employed a basis set of 30 and 60 functions for the harmonic and anharmonic double-well systems, respectively. Depending on the value of the system–bath coupling λ, we vary the truncation number of hierarchy from 6 to 12 to achieve the convergent results. To properly characterize the bath correlation function, 1 (2) Padé terms are included in the bath correlation function for small (large) values of γ. To facilitate the propagation of eq 33, we use a GPU (graphic processing unit) implementation of the BLAS (basic linear algebra subprograms) package (cuBLAS). The numerical integration of eq 33 is performed on a NVIDIA Tesla K40 GPU using a fourth-order Runge–Kutta method with a (dimensionless) time step of 0.002.

### 5.1 Harmonic Oscillator

Obviously, the system–bath dynamics of such a system can be solved analytically and similar harmonic systems have indeed been studied.^50−58^ Furthermore, as shown in Appendix B, the dynamics generated by the momentum coupling Hamiltonian of the present work and by the standard bilinear coordinate coupling Hamiltonian are in fact equivalent for harmonic systems. For example, the Laplace transform of the mean coordinate,  can be evaluated in the classical limit as follows (cf. refs (36 and 65)):
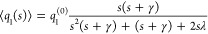
37The inversion of this expression requires the solution of the cubic equation and cannot be given in the compact form. In the Markovian limit (λ = *γλ*_γ_, γ → *∞*, λ_γ_ = const)
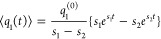
38where
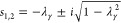
39For weak to moderate damping (λ_γ_ < 1), for example, ⟨*q*_1_(*t*)⟩ exhibits oscillations with a damping rate λ_γ_ and frequency  which decreases with λ_γ_.

Nevertheless, to set up the stage, we start from the study of harmonic system and take *U*_1_(*q*_1_) = ω_1_*q*_1_^2^/2. We will adopt the system harmonic oscillator frequency ω_1_ for the construction of dimensionless variables: Frequencies and energies are expressed in units of ω_1_, while time is expressed in units of 2π/ω_1_. In the calculations, we set the inverse temperature to *βω*_1_ = 0.5, vary system–bath coupling from weak (λ = 0.1ω_1_) through medium (λ = 0.4ω_1_) to relatively strong (λ = ω_1_) as well as consider almost Markovian bath (γ = 3ω_1_) and non-Markovian bath (γ = ω_1_).

**Figure 1:**
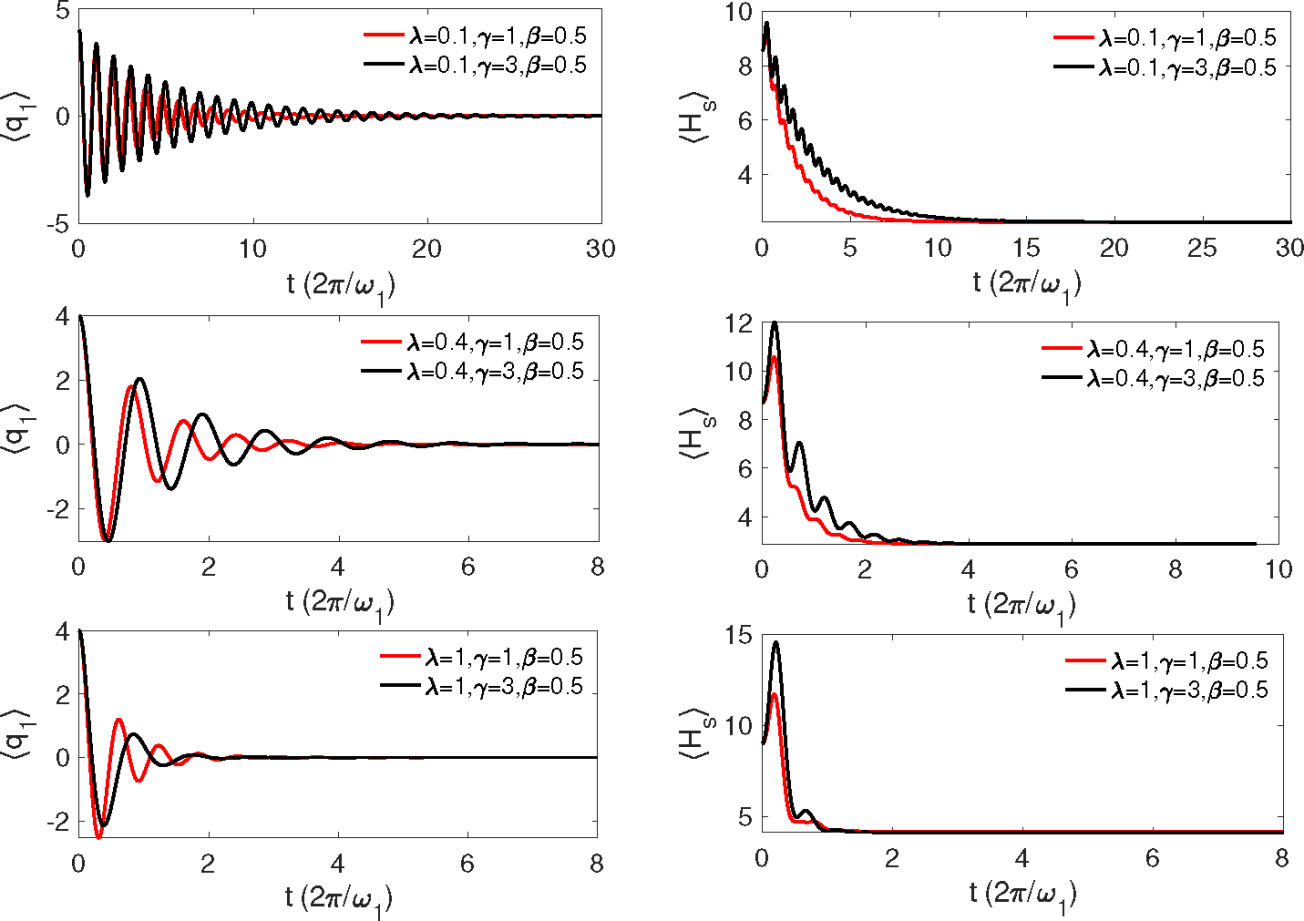
Expectation value of the position ⟨*q*_1_(*t*)⟩ (left column) and system energy ⟨*H*_S_(*t*)⟩ (right column) for different system–bath couplings λ and inverse bath memory times γ indicated in the panels. The inverse temperature is *βω*_1_ = 0.5.

The results of the simulations are presented in Figure 1 for ⟨*q*_1_(*t*)⟩ (left column) and ⟨*H*_S_(*t*)⟩ (right column). As explained above, the damping rate of the oscillations in ⟨*q*_1_(*t*)⟩ is controlled by the system–bath coupling λ and the period of these oscillations increases with λ. The bath memory also has visible impact on the ⟨*q*_1_(*t*)⟩ dynamics. As for the energy relaxation (right panels) two effects are to be pointed out. First, oscillations and their decay rate are faster for ⟨*H*_S_(*t*)⟩ than for ⟨*q*_1_(*t*)⟩. This is a direct consequence of the Gaussian nature of the harmonic oscillator dynamics, which requires that all higher-order correlation functions should be expressed as products of the lowest-order correlation functions. This means that, approximately, ⟨*H*_S_(*t*)⟩ should oscillate and decay twice faster than ⟨*q*_1_(*t*)⟩ (see, e.g., refs (65 and 66)), as indeed observed in Figure 1. Second, ⟨*H*_S_(*t*)⟩ initially increases with time. This is a consequence of the nonequilibrium preparation of the system according to eq 35, which results in the initial transfer of energy from the bath to the system (cf. refs (67 and 68)).

### 5.2 Double Well Potential

**Figure 2:**
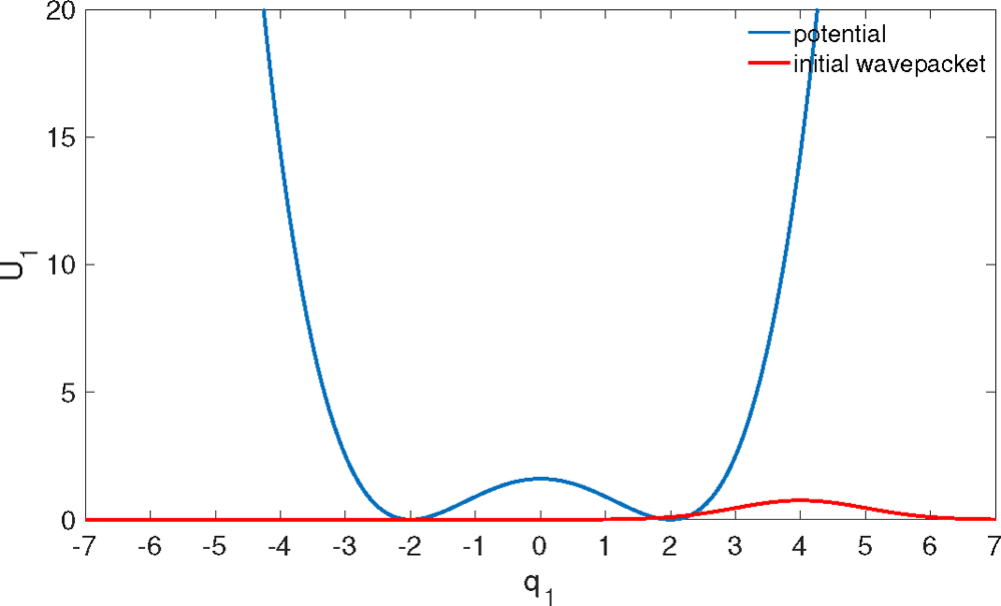
Double well potential energy of the system (blue curve) and the initial wavepacket (red curve).

To study the nonlinear dynamics, we chose the system Hamiltonian with a symmetric double well potential
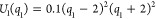
40(we set *ℏ* = μ_1_ = 1 for the construction of dimensionless variables). Figure 2 shows the corresponding potential energy function (blue curve) and the initial wavepacket (red curve).

**Figure 3:**
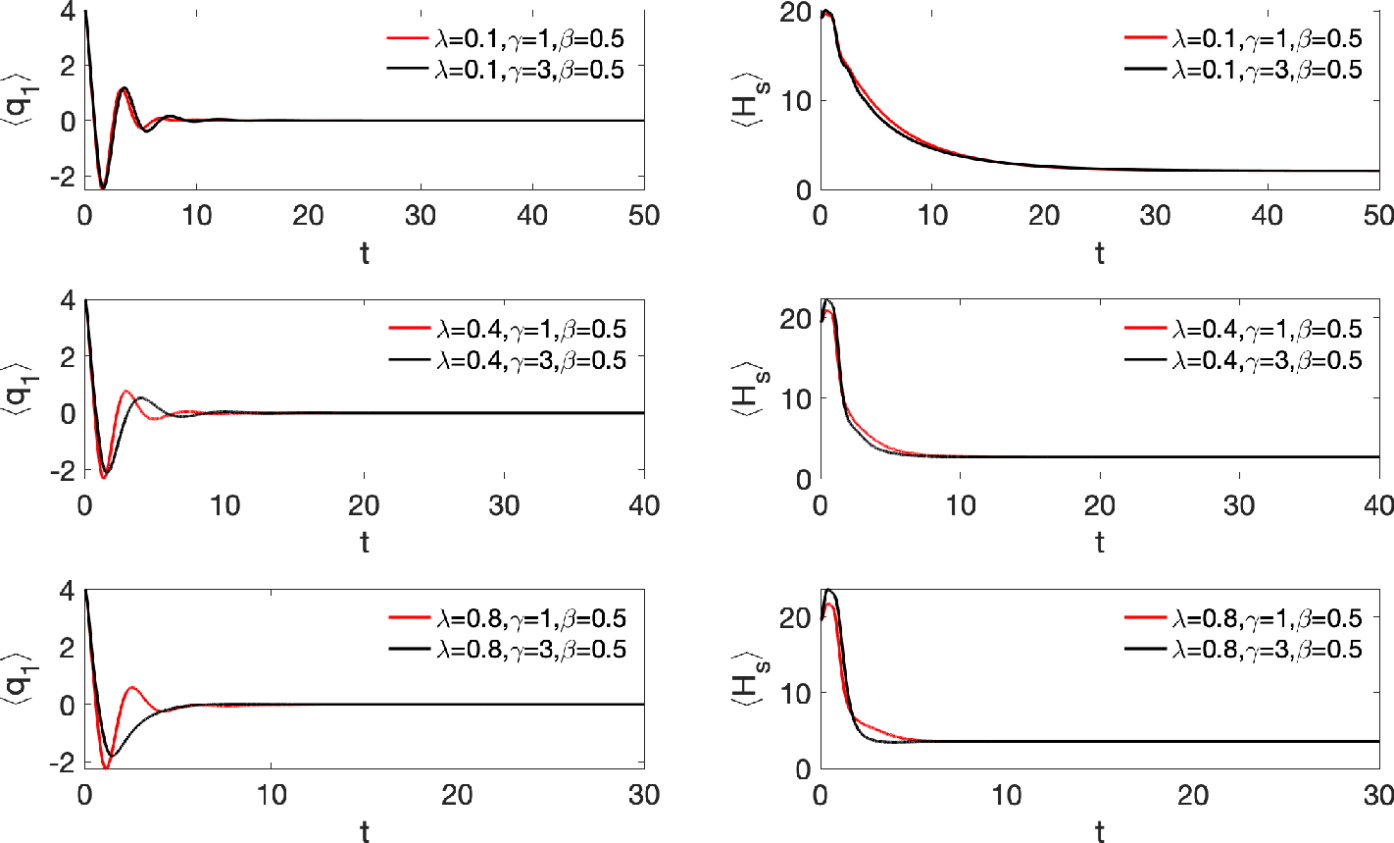
Expectation value of the position ⟨*q*_1_(*t*)⟩ (left column) and and system energy ⟨*H*_S_(*t*)⟩ (right column) for different system–bath couplings λ and inverse bath memory times γ indicated in the panels. The inverse temperature is β = 0.5.

The simulated ⟨*q*_1_(*t*)⟩ (left column) and ⟨*H*_S_(*t*)⟩ (right column) are presented in Figure 3 for various parameters of the model indicated in the figure. Nonlinearity has a drastic effect on the relaxation process: both ⟨*q*_1_(*t*)⟩ and ⟨*H*_S_(*t*)⟩ reach equilibrium much faster than in the harmonic case, and their dynamics is much less oscillatory (cf. ref (69)). In addition, non-Markovian effects are much less pronounced in the present case. With the preparation of the initial wavepacket shown in Figure 2, ⟨*q*_1_(*t*)⟩ and ⟨*H*_S_(*t*)⟩ of Figure 3 do not reveal any indication of the energy exchange between the two minima of the double-well potential, which may be manifested as an extra beating caused by the tunneling splitting. There are two reasons for that. First, mean positions and energies are not proper observables which can reveal this splitting. Second, the initial wavepacket is much closer to the right potential well (see Figure 2). Hence it is mostly nonlinearity of the problem which causes faster dephasing and relaxation of the wavepacket while the double-well nature of the potential is of secondary importance for the simulations presented in Figure 3. It should also be noted that equilibration time of the linear system is independent of the system’s preparation. For example, eq 37 shows that the details of the initial preparation (*q*_1_^(0)^) enter the expression for ⟨*q*_1_(*t*)⟩ just as a factor. This is no longer the case for nonlinear systems (see, e.g., ref (70)) and the study of different preparations of nonlinear systems is currently in progress.

## 6 Summary

We have demonstrated that, for a rather broad class of models, the Hamiltonian of the system nonlinearly interacting with a harmonic bath via coordinate coupling can be equivalently mapped into the Hamiltonian of the system bilinearly coupled to the bath via momentum coupling. This finding opens up the opportunity to scrutinize dynamics and relaxation processes in such systems via the numerically accurate HEOM machinery.

In the main body of the paper, we have studied the simplest situation, where a particle of interest is nonlinearly coupled to a harmonic chain. The reduction of the nonlinear coordinate coupling to bilinear momentum coupling for other classes of systems has been done in Appendix C. The present approach can also be extended toward systems coupled to several baths at different temperatures and toward systems coupled to each other via a harmonic bath. Other generalizations are also possible.

Finally, we mention that the momentum-momentum coupling Hamiltonian considered in this work is not limited to molecular chains, but it is a fundamental ingredient of any realistic model of vibrational energy redistribution processes.^45,71,72^ This envisages new applications of the HEOM methodology to realistic Hamiltonian models beyond the linear system–bath coupling.^73,74^

## Appendix A: Transformation of the Momentum Coupling Hamiltonian to the Coordinate Coupling Hamiltonian

Let us start from the total Hamiltonian of eq 7. For our purposes, it is elucidative to reinstall dimensional bath momenta and positions

41

where *M*_α_ is a mass associated with the αth bath oscillator. With these variables, the bath Hamiltonian of eq 19 is written as

42

and the system–bath coupling Hamiltonian of eq 20 assumes the form

43

(*C*_α_ has the unit of inverse mass).

Despite the fact that the transformation of eqs 2 and 3 is not canonical, the Hamiltonian of eq 7 is an absolutely legitimate quantum Hamiltonian which has a well-defined classical counterpart as well as a classical Lagrangian. The classical Lagrangian corresponding to the Hamiltonian of eq 7 is given by the expression

44

From the Euler–Lagrange equations we obtain the equations of motion

45

Following ref (75), we introduce a new set of coordinates given by

46

Here, *y*_α_ has the unit of momentum. In the new variables, the Lagrangian assumes the form

47

It generates the equations of motion

48

We further define the variables

49

which have the unit of position. Then eq 47 is transformed to

50

where *D*_α_ = *C*_α_*M*_α_Ω_α_ has the unit of angular frequency. The corresponding Hamiltonian is

51

This Hamiltonian differs from the original Hamiltonian of eqs 7, 8, 42, and 43 in two aspects. First, it contains the counter term in the system–bath coupling. Second, the momentum system–bath coupling is replaced by the coupling which is linear in the bath positions *R*_α_. In general, a possibility of such transformation is rooted into foundations of classical Hamiltonian mechanics: Labeling canonically conjugated variables as positions and momenta does not have a deep physical meaning, because a simple canonical transformations swaps positions and momenta.^76^

We can apply the standard path-integral machinery to the Hamiltonian of eq 51. Then, assuming factorized initial conditions, the reduced system density matrix is expressed in the standard path integral form^75^

52

where the influence functional reads

53

Here

54

and

55

Then, differentiation of eq 52 with respect to time (see, e.g. ref (3)) yields the HEOM of eq 33.

The equivalence of baths with position and momentum couplings demonstrated above indicates that the corresponding spectral densities of the baths, *J*^(*q*)^(ω) and *J*^(*p*)^(ω), can be chosen by invoking similar physical arguments and have similar physical meaning. *J*^(*q*)^(ω) for the harmonic chain model with nearest-neighbor coupling can be explicitly derived for the Rubin model,^35,36^ in which all chain oscillators are identical. In this case

where ω_*R*_ is the characteristic frequency. In the present work, we wish to adopt a more general description and choose to model *J*^(*p*)^(ω) by the Drude spectral density of eq 29. This spectral density is commonly used in the conventional position-coupling HEOM to describe a harmonic non-Markovian bath with the exponential memory function.

## Appendix B: Coordinate Bath vs Momentum Bath

### Coordinate Bath

Let us consider the standard system–bath Hamiltonian with bilinear coordinate coupling

56

This Hamiltonian generates the following (classical or quantum) equations of motion:

57

Expressing momenta through coordinates, we obtain

58

Hence

59

or, equivalently

60

We thus arrive at the generalized Langevin equation^36^

61

in which

62

is the memory kernel, η_*q*_(0) is the bath-induced renormalization of the system potential (it can be incorporated into the counter term in the bath Hamiltonian), and

63

is the Gaussian stochastic force. In the classical limit, the exponential memory kernel η_*q*_(*t*) = λ exp(−*γt*) corresponds to the Drude spectral density of eq 29.

### Momentum Bath

The momentum-coupling Hamiltonian of the present work reads

64

The corresponding equations of motion are as follows

65

66

Expressing momenta through coordinates, we obtain

67

Hence

68

and we arrive at the generalized Langevin equation of the kind

69

where

70

is the memory kernel, η_*p*_(0) is the bath-induced renormalization of the system mass (it also can be incorporated into the counter term in the bath Hamiltonian), and

71

As distinct from the generalized Langevin eq 61 generated by the coordinate coupling Hamiltonian, the Langevin equation of eq 69 contains system coordinate and momentum operators. The latter can be expressed through the system coordinates via the second part of eqs 65, but the resulting generalized Langevin equation will contain the derivative of the system potential and (in general) will be highly nonlinear and difficult to interpret (cf. ref (52)). It is only in the case of harmonic potential when

72

and

73

Up to the redefinition of the system–bath coupling constants *g*_*a*_^(*q*)^ and *g*_*a*_^(*p*)^, eqs 61 and 73 are equivalent.

## Appendix C: Generalizations of the Chain Model

### Particle in an External Potential

Let us add an external potential *V*(*X*_1_) to the Hamiltonian of eq 1:

74

Introduce

75

Then the original momenta *P*_*k*_ are connected to the new momenta *P* ≡–*iℏd*/*dQ* and *p*_*k*_ ≡–*iℏd*/*dq*_*k*_ as follows:

76

Then

77

(*p*_*N*_ ≡ 0).

Now we recast eq 77 in the system–bath form of eq 7 where

78

while *H*_SB_ and *H*_B_ are given by eqs 9 and 10, respectively. The entire derivation then goes as in section 3. In fact the system–bath interaction is included into the extended system Hamiltonian of eq 78. These equations could be of interest for exploring quantum thermodynamics in the case of strong system–bath coupling.^77^

### Two Particles Nonlinearly Coupled to a Chain of Harmonic Oscillators

Let us consider the starting Hamiltonian of eq 1 in which *U*_1_(*X*_2_ – *X*_1_) and *U*_*N*–1_(*X*_*N*_ – *X*_*N*–1_) are certain nonlinear potentials. This corresponds to the situation where the first and the last particle of the chain interact nonlinearly with their neighbors, while the latter are connected to each other via a harmonic chain (quantum dynamics of similar systems has recently been studied via tensor-train methods^46^). This model is described by the Hamiltonian of eq 6 in which *U*_1_(*q*_1_) and *U*_*N*–1_(*q*_*N*–1_) are arbitrary and

79

The Hamiltonian of this problem can again be recast in the system–bath form of eq 7 with bilinear momentum coupling where

80

is the system Hamiltonian

81

is the system–bath momentum coupling Hamiltonian, and

82
